# Pirfenidone treatment attenuates fibrosis in autosomal dominant polycystic kidney disease

**DOI:** 10.1101/2025.08.25.672225

**Published:** 2025-08-29

**Authors:** Viji Remadevi, Abeda Jamadar, Meekha M Varghese, Sumedha Gunewardena, Darren P Wallace, Reena Rao

**Affiliations:** 1 Jared Grantham Kidney Institute, University of Kansas Medical Center, Kansas City, KS.; 2 Division of Nephrology, Department of Medicine, University of Kansas Medical Center, Kansas City, KS.; 3 Cell Biology and Physiology Department, University of Kansas Medical Center, Kansas City, KS, USA

**Keywords:** Myofibroblasts, α-smooth muscle actin, Pirfenidone, Fibrosis, Extracellualr matrix, Therapy

## Abstract

**Introduction:**

Autosomal dominant polycystic kidney disease (ADPKD) is characterized by the formation of fluid filled cysts, progressive fibrosis and chronic inflammation, often leading to kidney failure. Renal fibrosis in ADPKD is primarily driven by myofibroblast activation and excessive extracellular matrix (ECM) accumulation, which contribute to disease progression. Here we investigated the therapeutic potential of pirfenidone, an antifibrotic drug, on myofibroblast activity, ECM production, and ADPKD progression.

**Methods:**

Primary cultures of myofibroblasts from human ADPKD kidneys were treated with pirfenidone *in* vitro, and cell proliferation, migration, contractility and changes in ECM production were measured. *In vivo*, the effect of pirfenidone on cyst growth, fibrosis and renal function were determined in the Pkd1^RC/RC^ male mouse model of ADPKD and wild type controls.

**Results:**

Analysis of single-nucleus RNA sequencing data of human ADPKD kidneys revealed that fibroblasts are a primary source of fibrous and cell-adhesive ECM, with higher ECM gene expression compared to normal human kidneys. Treatment of human ADPKD renal myofibroblasts with pirfenidone led to reduced ECM gene expression, cell proliferation, migration and contractility. *In vivo*, pirfenidone treatment in Pkd1^RC/RC^ mice reduced renal fibrosis, collagen deposition, myofibroblast accumulation, pro-fibrotic gene expression and decreased TGF-β/SMAD3 and mTOR signaling. While kidney cyst number remained unchanged, kidney size and cyst area were reduced, leading to improved kidney morphology and improved renal function in RC/RC mice.

**Conclusion:**

These findings suggest that pirfenidone mitigates renal fibrosis and preserves renal architecture in ADPKD, supporting its potential as a therapeutic strategy to inhibit fibrosis in ADPKD.

## Translational Statement

Autosomal dominant polycystic kidney disease (ADPKD) is characterized by cyst growth and progressive renal fibrosis, which contributes significantly to kidney function decline. Current therapies predominantly target cyst expansion but do not adequately address fibrosis. Our study demonstrates that myofibroblasts are the principal source of extracellular matrix (ECM) deposition in ADPKD kidneys and that pirfenidone, an FDA-approved antifibrotic drug for idiopathic pulmonary fibrosis, effectively reduces myofibroblast activation, ECM production, and key profibrotic signaling pathways both in human ADPKD cells and in a rodent model. Importantly, pirfenidone treatment also decreased kidney enlargement and cyst growth without nephrotoxicity. These findings support the repurposing of pirfenidone as a complementary therapeutic strategy in ADPKD to target fibrotic remodeling alongside cystic disease. Clinical trials investigating pirfenidone’s safety and efficacy in ADPKD patients could potentially slow disease progression and improve renal outcomes.

## INTRODUCTION

ADPKD is a common inherited monogenic disorder caused by mutations in the PKD1 or PKD2 genes. It is estimated that 42.6 per 100,000 persons in the United States, and over 12.5 million people worldwide are afflicted with ADPKD ^1, 2^. Progressive cyst growth in the kidneys and liver, often accompanied by interstitial fibrosis, inflammation, and hypertrophy are hallmarks of ADPKD ^3^. Among these features, renal fibrosis is a critical pathological component, and a major contributor to declining renal function and eventual progression to end-stage kidney disease (ESKD) ^4-7^.

Renal interstitial fibrosis involves excessive accumulation and reduced degradation of ECM proteins, leading to tissue scarring and disruption of normal kidney achitecture and function. As renal fibrosis progresses, it contributes to systemic complications such as hypertension, metabolic dysregulation and electrolyte imbalance. ADPKD kidneys show excessive accumulation, abnormal turnover and remodeling of the ECM, and activation of pro-fibrotic cell signaling pathways ^4-6^. Prior studies indicate that ECM accumulation also contributes to cyst growth *via* integrin signalling ^7, 8^. The primary producers of ECM in chronic kidney disease (CKD) are myofibroblasts, which are highly contractile α-smooth muscle actin (αSMA)- expressing cells ^9^. Our previous studies showed that renal cyst- lining epithelial cells drive myofibroblast activation, accumulation *via* paracrine signaling ^10^. Moreover, depleting myofibroblasts in a mouse model of ADPKD reduced renal fibrosis and cyst growth, showing their key pathogenic role ^11^.

Most ADPKD cases are diagnosed when cysts are already evident, and kidney function begins to decline, often alongside comorbidities like hypertension, except in individuals with a known family history ^12^. Importantly, Tolvaptan is approved for use in patients with advanced cystic disease (Mayo-Irazabal Classification 1D-1E) ^13^; however by this stage, the kidneys are already fibrotic, and continued ECM accumulation further compromises kidney function. Therefore, preventing or reversing renal fibrosis remains a key therapeutic objective in ADPKD.

Pirfenidone (5-methyl-1-phenyl-2-[1H]-pyridinone) is a pyridine derivative antifibrotic drug approved for the treatment of idiopathic pulmonary fibrosis (IPF) ^14^. It shows antifibrotic, anti-inflammatory and antioxidant effects across preclinical and clinical models, including fibrotic lesions in the heart, liver, skin, and pancreas ^15^. Pirfenidone is also kidney-protective in animal models of renal injury including unilateral ureteral obstruction, nephrotoxicity, diabetic nephropathy and anti-glomerular basement membrane glomerulonephritis, mainly by targeting TGF-β signaling and reducing ECM deposition ^16-18^.

Given the central role of fibrosis in ADPKD progression, we investigated whether pirfenidone could attenuate renal fibrosis in the well-established Pkd1^RC/RC^ mouse model of slowly progressive ADPKD. We also aimed to elucidate the underlying molecular mechanisms of its action, with a focus on ECM remodeling, myofibroblast activity and key signaling pathways. Our findings demonstrate that pirfenidone reduces myofibroblast activity and renal fibrosis, and may be a potential therapeutic drug for the treatment of established PKD.

## MATERIALS AND METHODS

### *In vivo* study

#### The ADPKD mouse model:

Pkd1^RC/RC^ (RC/RC) mouse ^10, 19^ is a slow progressing, adult, orthologous model of ADPKD carrying a temperature sensitive folding hypomorphic mutation (R3277C) in the Pkd1 gene. RC/RC and WT mice are on pure BALB/c background, and inbred. Male WT and RC/RC mouse littermates were treated with vehicle (5% DMSO+1% Hydroxymethyl cellulose) or pirfenidone (#HY-B0673, MedChemExpress, USA) (200mg/kg BWt., twice daily) by oral gavage between 8-9 AM and 4-5 PM, 6 days a week, from 4 to 6 months of age and sacrificed at 6 months of age. A power analysis conducted based our previous study ^20^ determined that using 9 mice will provide 80% statistical power to detect a 40% reduction in cyst area in RC/RC mice treated with drug compared to controls (significance level (α) 0.05).Mice from each litter were assigned to study groups as they reached 4 months of age. No randomisation was used. The investigator who performed the study and analysis was blinded to the identity of the mice. Mice were housed in a temperature controlled environment in a 12 hr light /12hr dark cycle. Experimental mice were placed on the same rack. All mice were sacrificed between 11:30 AM and 12:30 PM. Blood was collected and plasma isolated. Kidneys were weighed and flash frozen, or fixed in 4% paraformaldehyde. No adverse effect was observed and no mice or datapoints were excluded. All mouse related studies were approved by University of Kansas IACUC committee. ARRIVE guidelines were followed for animal studies ^21^.

#### H&E staining and quantification of cysts:

H&E staining was performed on 5μm thick kidney tissue sections and imaged using Nikon 80i upright microscope, Tokyo, Japan. Cyst area, cyst number and total kidney area were quantified using ImageJ, Madison WI, USA by an observer blinded to the sample’s identity ^22^.

#### Picros Sirius red staining:

Tissue fibrosis (collagen-1) was detected by staining tissue sections using Picros Sirius red staining kit (#PSR-2, Scy Tek Laboratories, Utah, USA) following the manufacturer’s protocol.

#### Blood-urea nitrogen level (BUN):

Plasma BUN was measured using commercially available QuantiChrom Urea Assay Kit (#DIUR-100, BioAssay Systems, Hayward, CA, USA) ^23^.

#### Western blot:

Kidneys were homogenized in SDS Laemmli buffer and run in 10% SDS-polyacrylamide agarose electrophoresis gels as described earlier ^24^. Primary antibodies for αSMA (#ab5694; Abcam, Cambridge, MA,USA), pSmad3 (#9520T), Smad3 (#9513S), pS6(#4858S) and S6(#2317S) from Cell signaling, Danvers, MA, USA; and GAPDH (#SC-32233; Santa Cruz Biotechnology, Inc, Dallas, TX, USA), as well as anti-mouse (#P0447) and anti-rabbit (#P0448) secondary antibodies from Dako (Santa Clara, CA, USA) were used. Immunoreactive proteins were detected using ECL reagent (Amersham, GE Healthcare, Buckinghamshire, UK).

#### Immunofluorescence:

Fixed and paraffin-embedded tissues sections were processed as previously described ^25^. Primary antibodies αSMA (#ab5694) from Abcam (Cambridge, MA, USA) and Collagen Type-1a (#203002 from MD Bioproducts, Oakdale, MN) were applied followed by incubation with secondary antibodies anti-Rabbit IgG Alexa fluor^@^ 488 and anti-Goat IgG Alexa fluor^@^ 594 from Invitrogen (Carlsbad, CA, USA). After incubation, tissue sections were washed, stained with DAPI, and mounted using Flour-G (Invitrogen, Carlsbad, CA, USA). Images were captured using a Nikon 90i upright microscope (Tokyo, Japan).

#### Quantitative real time PCR:

RNA was isolated from whole kidney lysate using trizol method and cDNA prepared with High-capacity cDNA reverse transcription kit (# 4368814, Applied Biosystems, Foster City, CA, USA). SYBR Green PCR master mix (# A25742, Applied Biosystems, Foster City, CA, USA) was used for QRT-PCR ^11^. Table-1 shows the primer sequences.

### *In vitro* studies

#### Primary culture human ADPKD myofibroblasts:

Cells were isolated from human ADPKD kidneys as described earlier ^20, 26^. Cells were used in their first passage and grown in DMEM:F12 media with 10% FBS and 1% Pen/Strep.

#### Study approval:

Human ADPKD kidney tissues were from the University of Kansas PKD Biomarkers and Biomedical core. Mycoplasma contamination was monitored using DAPI staining and confirmed using PCR based mycoplasma testing kit.

#### NRK-49F WT cells:

Rat renal fibroblasts, NRK-49F cells (#CRL-1570, ATCC^©^, Manassas, VA, USA) were grown in DMEM medium with 5% FBS and 1% Pen/Strep.

#### Cell viability and cell proliferation assays:

MTT assay was conducted to assess cell viability as described before ^27^. To measure cell proliferation by BrdU incorporation assay ^27^, human ADPKD renal myofibroblasts grown on coverslips were serum starved overnight followed by release into media containing 0.2% FBS and treated with pirfenidone or vehicle. After 24h treatment, cells were incubated with 3μg/ml BrdU (#10280879001, Millipore Sigma, Burlington, MA) for 3h, and cell proliferation was measured as BRDU/DAPI expressed as %.

#### Migration assay:

Confluent monolayers of human ADPKD renal myofibroblasts were treated with 5μg/ml mitomycin c (# M7949; Sigma Aldrich; MO; USA) for 2h; washed twice with PBS and a single scratch (wound) was created manually across the monolayer using a sterile pipette tip, and cell debris washed off using PBS. The cells were treated with vehicle or pirfenidone. The wound was imaged at regular intervals using Phase contrast microscope until the wounds in any one study group closed 100%, and wound closure was quantified ^28^.

#### Gel contractility assay:

Human ADPKD myofibroblasts were trypisinized and resuspended in complete medium, and mixed with rat-tail collagen type-1 (pH 7.4) (# 354236, Corning, Glendale, Arizona). 500μl of collagen/cell mixture containing 1.5 X 10^5^ cells was dispensed into 24-well cell culture plates coated with 0.2% BSA. The mixture was incubated to polymerize at 37°C in a CO_2_ incubator for 1hr. After polymerization, the gels were detached gently from the sides and were supplemented with serum free medium. The gels were imaged at different time points and the gel area was calculated using image-J software.

#### Single-nucleus RNA sequencing:

**Figure 1:**
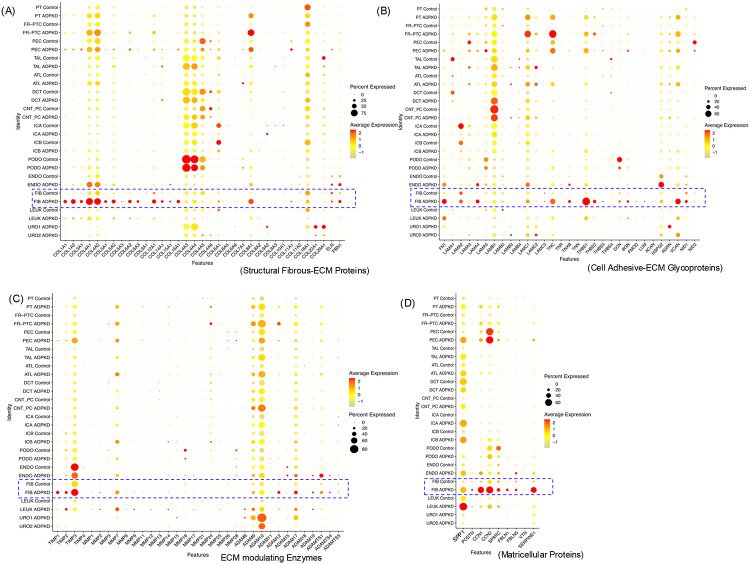
Cell type specific expression of ECM-related genes in human ADPKD or normal control kidneys. Dot plots representing the snRNA-seq Kidney interactive transcriptomics (KIT) dataset illustrates the expression patterns of genes that are enriched within specific cell subtypes from ADPKD (n=8) and normal control (n=6) human kidney samples. (A) Gene expression of structural fibrous ECM proteins, (B) cell adhesive ECM-glycoproteins (C) ECM-modulating enzymes, and (D) matricellular proteins. The size (diameter) of each dot reflects the percentage of cells expressing the gene, while the color intensity indicates the average expression level compared to all cell types.

The snRNA-Seq data corresponding to Fig 1 was obtained from Muto, Y. et al.^29^ (GSE185948) https://humphreyslab.com/SingleCell/search.php. The bioinformatics analysis of this data was performed in line with the description given by the authors in their Methods section. We recreated the exact R environment used by the authors in order to obtain results comparable to theirs for genes of our interest presented in the dot-plots.

#### Statistics:

Values were expressed as mean ± standard error (SEM) for *in vivo* studies, and mean ± standard deviation for *in vitro* studies. The data were analyzed by two-tailed unpaired *t*-test with Welch’s correction, or one-way ANOVA followed by Tukey`s multiple comparisons test. P≤0.05 was considered statistically significant. Analysis was done using GraphPad Prism software Version 9.1.0 (216) (GraphPad Software, Inc., La Jolla, CA, USA).

## RESULTS

### Myofibroblasts are a predominant source of ECM in human ADPKD kidneys.

Myofibroblasts are the major producers of ECM in chronic kidney disease ^30^. However in ADPKD kidneys, the specific renal cell types that produce ECM, the composition and the relative levels of ECM expressed by these cell types compared to normal kidneys remain poorly defined. We analyzed the publicly available Kidney Interactive Transcriptomics (KIT) single-nucleus RNA sequencing (snRNA-seq) database comparing human normal kidneys (n=5) and ADPKD kidneys (n=8) ^29^. A cutoff of gene expression of >=1 was applied to identify genes actively transcribed in the cell clusters. Among the structural fibrous ECM proteins, we detected gene expression of 21 collagen subtypes, as well as elastin (ELN) and fibrillin-1 (FBN1) in the kidney cell clusters (Fig 1A). COL4A subtypes COL4A3 and COL4A4 exhibited the high expression levels among all collagens, and were predominantly expressed in the podocytes (Fig 1A). Compared to other renal cell types, ADPKD fibroblast cluster was the major source of 13 collagen subtypes, including COL1A1, COL1A2, COL3A1, COL4A1, COL4A2, COL5A1, COL5A2, COL6A2, COL6A3, COL12A1, COL14A1, COL15A1, and COL16A1 (Fig 1A). Notably, the expression of the above mentioned collagens, along with COL8A1 and FBN1 were elevated in the fibroblast cluster of ADPKD kidneys compared to normal control kidneys (Fig 1A). Non-fibroblast cell types expressed the structural fibrous ECM proteins including, COL4A3 and COL4A4, which were enriched in podocytes, while COL4A5, COL8A1, and COL18A1 were primarily expressed in tubular epithelial cells (Fig 1A).

Of the 18 genes of cell adhesive ECM glycoproteins expressed in the ADPKD kidneys, ADPKD fibroblast cluters were the major expressors of 9 genes including fibronectin (FN1), laminin subunit α4 (LAMA4), tenascin-XB (TNXB), thrombospondin-1 (THBS1), THBS2, biglycan (BGN), versican (VCAN), and nidogen-1 (NID1), compared to other renal cell types (Fig 1B). The above mentioned glycoproteins, along with tenascin-C (TNC), LAMC1, LAMC2, and perlecan (HSPG2) showed high expression in ADPKD fibroblasts relative to normal control kidney fibroblasts (Fig 1B).

We also analyzed the expression of tissue inhibitors of metalloproteinases (TIMPs) and matrix metalloproteinases (MMPs), which directly regulate ECM remodeling and turnover, as well as ADAMs, which indirectly influence ECM remodeling through growth factor shedding, myofibroblast differentiation, and inflammatory signaling^5^. Expression of TIMP1, TIMP2, TIMP3, ADAM12 and ADAM17 genes were higher in fibroblasts compared to other cell types (Fig 1C). The above mentioned genes, and MMP7, ADAM10 and ADAMTS1 exhibited higher expression levels in fibroblast clusters of ADPKD kidneys compared to normal control kidneys (Fig 1C).

Although matricellular proteins are non-structural components of the ECM, they play critical roles in modulating cell-matrix signaling and regulating myofibroblast differentiation, thereby contributing to ECM remodeling and fibrosis. Several matricellular proteins, including osteopontin (SPP1), cellular communication network factors (CCN1 and CCN2), osteonectin (SPARC), fibulin-1 (FBLN1), FBLN5 and SERPINE1 exhibited higher expression in fibroblasts clusters from ADPKD kidneys compared to normal control kidneys (Fig 1D). Notably, CCN1 and SERPINE1 were among the most highly expressed in ADPKD fibroblasts relative to all other renal cell types.

### Pirfenidone treatment reduced the expression of collagens in human ADPKD renal myofibroblasts.

**Figure 2:**
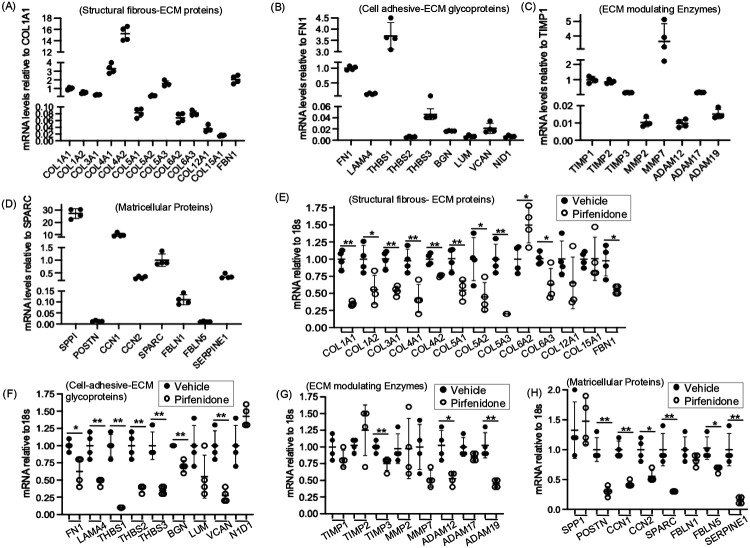
Effect of pirfenidone treatment on expression of ECM-related genes in human ADPKD renal myofibroblasts. (A) In primary culture human ADPKD myofibroblasts, relative gene expression of structural fibrous ECM proteins, (B) cell adhesive ECM-glycoproteins, (C) ECM-modulating enzymes, and (D) matricellular proteins. (E) Effect of pirfenidone (0.5mg/ml for 24h) or vehicle treatment on mRNA levels of structural fibrous ECM proteins, (F) cell adhesive ECM-glycoproteins (G) ECM-modulating enzymes, and (H) matricellular proteins in human ADPKD myofibroblasts. *P<0.05, **P<0.01 by two-tailed unpaired t-test with Welch’s correction.

To validate the findings from the snRNA-seq analysis, we examined the expression of ECM and related proteins in primary myofibroblast cell cultures derived from human ADPKD kidneys. Most structural fibrous ECM proteins identified in ADPKD fibroblasts by snRNA-seq were detected in these ADPKD myofibroblasts, with COL4A1 and COL4A2 showing the highest expression levels (Fig 2A). Among the cell-adhesive ECM glycoproteins, FN1, LAMA4, and THBS1 were prominently expressed (Fig 2B). Similarly, ECM-remodelling enzymes TIMP1, TIMP2, TIMP3, MMP7, ADAM17, and matricellular proteins CCN1, CCN2, SPARC, FBLN1 and Serpine-1 were predominantly expressed in ADPKD myofibroblasts (Fig 2C, D).

To evaluate the effect of pirfenidone on ECM production, we treated human ADPKD myofibroblasts with 0.5 mg/ml pirfenidone for 24h hours and assessed mRNA expression levels of ECM components. Pirfenidone treatment significantly reduced gene expression of most ECM proteins except COL12A1, COL15A1, LUM and NID1, and COL6A2, which showed increase compared to vehicle treatment (Fig 2E,F). Among the ECM-modulating enzymes, pirfenidone reduced the expression of only TIMP3, ADAM12, and ADAM19 (Fig 2G). Additionally, all examined matricellular proteins except FBLN1 showed reduced expression in response to pirfenidone (Fig 2H). Collectively, these data indicate that pirfenidone significantly attenuates the expression of ECM and ECM-related genes in human ADPKD renal myofibroblasts.

### Pirfenidone reduced proliferation, migration and contractility of human ADPKD renal myofibroblasts.

**Figure 3:**
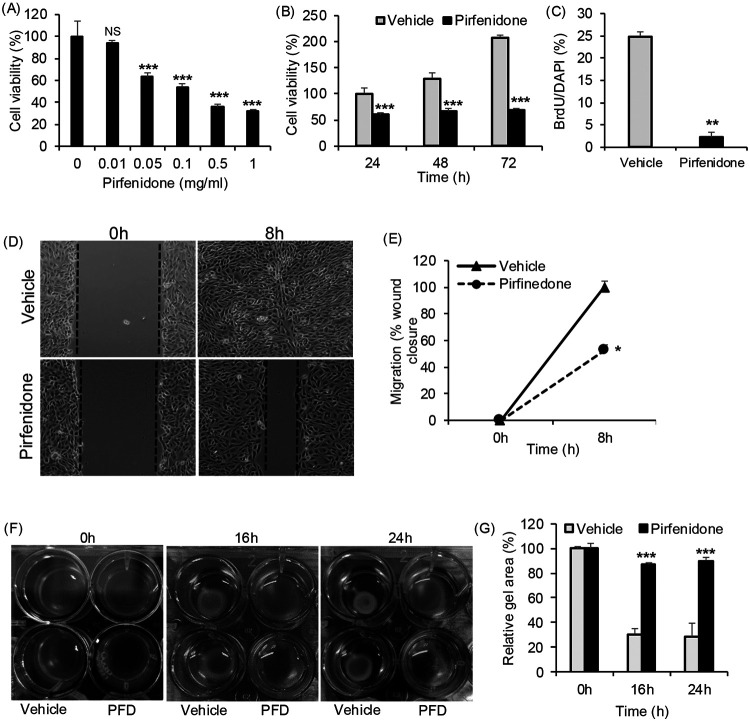
Effect of pirfenidone on human ADPKD myofibroblast function. (A) In human ADPKD myofibroblasts, cell viability assessed by MTT assay after pirfenidone treatment for 24hrs. (B) Time course of cell viability in cells treated with 0.5mg/ml pirfenidone on myofibroblast cell viability. (C) Cell proliferation measured by BrdU assay after treatment with vehicle or pirfenidone (0.5mg/ml for 24hrs). (D) Cell migration assay in cells treated with vehicle or pirfenidone (0.5mg/ml) (E) Quantification of migration. (F) Gel contractility assay in cells treated with vehicle or pirfenidone (0.5mg/ml). (G) Area of the collagen lattice. *P<0.05, **P<0.01, ***P<0.001 by two-tailed unpaired t-test with Welch’s correction.

Pirfenidone treatment time and dose-dependently reduced human ADPKD myofibroblast cell viability (Fig 3A,B), and reduced cell proliferation indicated by a ten-fold reduction in BrdU incorporation compared to vehicle-treated controls (Fig 3C). In addition, pirfenidone markedly reduced the migratory capacity of ADPKD myofibroblasts (Fig 3D, E). In a scratch-wound healing assay using mitomycin-pretreatment to block cell proliferation, vehicle-treated myofibroblasts closed the wound within 8 hours, whereas pirfenidone-treated cells closed only ~50% of the wound area over the same time period (Fig 3D, E).

Myofibroblasts express αSMA, contributing to their contractile phenotype and driving tissue distortion and disease progression in solid organs^31^. When cultured in collagen gels, vehicle treated ADPKD myofibroblasts contracted the gel within 16 hours, compared to which, pirfenidone-treated myofibroblasts showed 60% less gel contraction (Fig 3F, G).

### Pirfenidone treatment reduced renal fibrosis in RC/RC mice.

**Figure 4:**
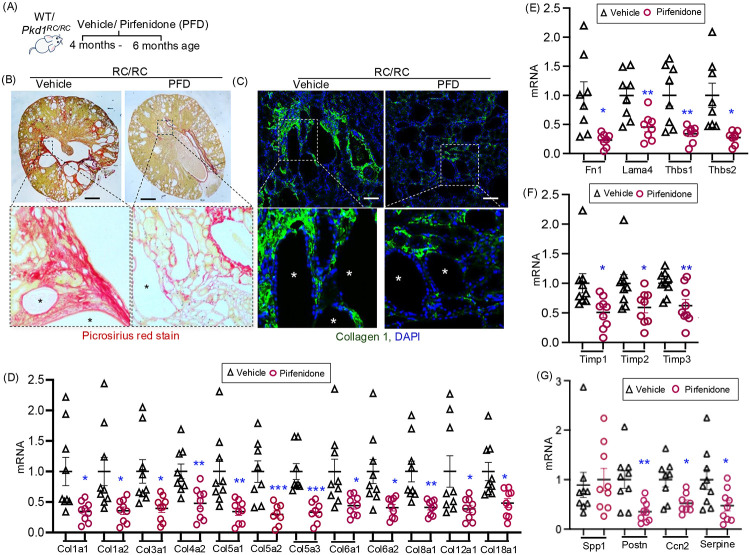
Effect of pirfenidone on fibrosis in RC/RC mouse kidneys: (A) Schematic overview of study protocol. WT and Pkd1^RC/RC^ (RC/RC) mice were treated with vehicle or pirfenidone (200mg/kg, twice daily, 6 days per week by oral gavage for 2 months). (B) Sirius Red staining of RC/RC mouse kidney tissue (Scale bar 1mm). (C) RC/RC mouse kidney tissue immunostained for Collagen 1A (green) and DAPI (nuclei, blue) (Scale bar 100μm). (D) Renal mRNA levels of structural fibrous ECM proteins, (E) cell adhesive-ECM glycoproteins, (F) ECM-modulating enzymes and (G) matricellular proteins in RC/RC mouse renal tissues. *P<0.05, **P<0.01, ***P<0.001 by two-tailed unpaired t-test with Welch’s correction. Star symbols in figures B and C represent cysts.

To determine the effect of pirfenidone on renal fibrosis in ADPKD, male Pkd1^RC/RC^ (RC/RC) mice were treated with vehicle or pirfenidone (200mg/kg, twice-a-day by oral gavage), from 4 months of age to 6 months of age and sacrificed (Fig 4A). Pirfenidone treated RC/RC mice had reduced renal collagen-1 expression, as demonstrated by decreased Picrosirius Red staining and immunofluorescent labeling for collagen-1, compared to vehicle-treated RC/RC controls (Fig 4B, C). At the transcript level, pirfenidone treatment significantly decreased the expression of multiple collagens, including Col1a1, Col1a2, Col3a1, Col4a2, Col5a1, Col5a2, Col5a3, Col6a1, Col6a2, Col8a1, Col12a1, and Col18a1 (Fig 4D). While Col4a1 and Col15a1 were also detected, their expression levels were not significantly altered (Supplemental Fig 1A). Additionally, the expression of other ECM-related genes such as Fn1, Lama4, Thbs1, and Thbs2 were significantly reduced in pirfenidone-treated RC/RC kidneys compared to vehicle-treated controls (Fig 4E).

Pirfenidone treatment also reduced renal mRNA levels of Timp1, Timp2 and Timp3 in RC/RC mice compared to vehicle-treated controls (Fig 4F). In contrast, the expression of Mmp2, Mmp7, and Mmp9 showed no significant differences between pirfenidone- and vehicle-treated RC/RC mice (Supplemental Fig 1B). Among the matricellular proteins, Postn1, Ccn2 and Serpine-1 showed significant decrease in pirfenidone treated RC/RC kidneys compared to vehicle treatment (Fig 4G).

### Pirfenidone treatment reduced renal myofibroblasts, and TGFβ and mTOR-mediated cell signaling in RC/RC mice.

**Figure 5:**
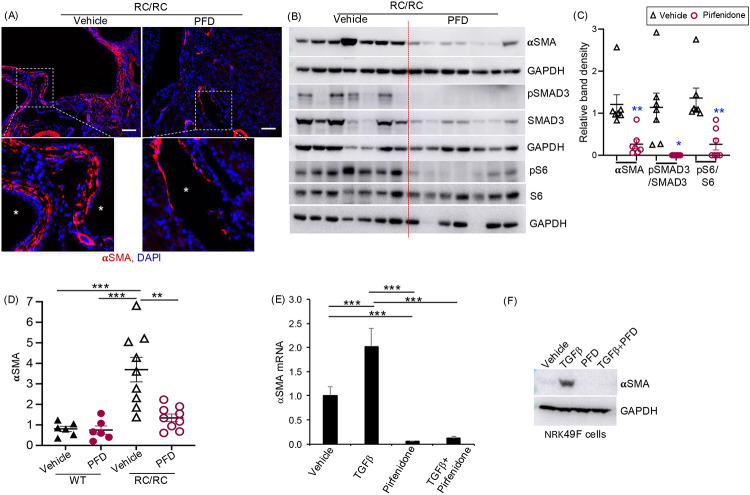
Effect of pirfenidone on myofibroblast activation in RC/RC mouse kidneys: (A) Representative images of Pkd1^RC/RC^ mice immunostained for αSMA (red) and nuclei stained using DAPI (blue) (Scale bar 100μm). (B) Western blot analysis of the whole kidney tissue lysate. (C) Densitometry of immunoblots. (D) αSMA mRNA levels relative to 18S in RC/RC mouse kidney tissue. (E) mRNA level analysis of αSMA expression in NRK-49F cells treated with TGFβ and pirfenidone for 24h. (F) Western blot analysis of NRK-49F cells treated with TGFβ (2ng/ml) and pirfenidone (0.5mg/ml) for 24h. *P<0.05, **P<0.01, ***P<0.001 by two-tailed unpaired t-test with Welch’s correction. Star symbols in figures B and C represent cysts.

We examined the effect of pirfenidone treatment on the expression of αSMA, a well-established myofibroblast marker. In the vehicle treated RC/RC mouse kidneys, αSMA immunostaining revealed dense layers of myofibroblasts surrounding the cyst-lining epithelium. Pirfenidone treatment exhibited markedly reduced αSMA expression in the RC/RC kidney (Fig 5A), indicating a reduction in myofibroblasts. Quantitative analysis confirmed a significant decrease in both αSMA protein expression (Fig 5B,C) and Acta2 (αSMA) mRNA levels (Fig 5D) in pirfenidone-treated mice compared to vehicle-treated controls.

Immunoblot analysis revealed that pirfenidone-treated RC/RC mouse kidneys had reduced pSMAD3/SMAD3 levels, indicating attenuation of TGFβ signaling (Fig 5B,C). Pirfenidone treatment also significantly lowered the pS6/S6 ratio, suggesting a suppression of mTOR signaling (Fig 5B,C).

TGFβ signaling is a key driver of fibroblast-to-myofibroblast differentiation and the excessive production of ECM. Supporting our *in vivo* findings, TGFβ treatment of cultured NRK-49F rat renal fibroblasts significantly induced Acta2 (αSMA) mRNA (Fig 5E) and protein expression (Fig 5F), which were completely abolished when simultaneously treated with pirfenidone (Fig 5E,F).

### Pirfenidone treatment reduced renal cyst growth in RC/RCmice

**Figure 6:**
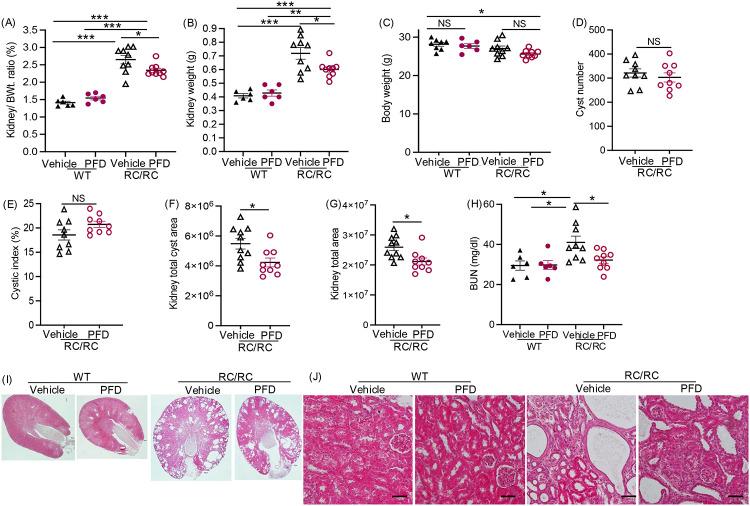
Effect of pirfenidone on cyst growth in RC/RC mice: (A) Two kidneys to body weight ratios (%). (PFD = Pirfenidone) (B) Total kidney weight. (C) Body weight. (D) Cyst number (E) Cystic index (%). (F) Kidney total cyst area. (G) Kidney total area. (H) BUN (I) Representative images of H&E staining (Scale bar 1mm), and (J) Magnified images (Scale bar 100μm). *P<0.05, **P<0.01, ***P<0.001 by two-tailed unpaired t-test with Welch’s correction for D,E,F,and G; and by ordinary one-way ANOVA with Tukey’s multiple comparison test in A,B,C and H.

Pirfenidone treatment significantly reduced kidney-to-body weight ratio (Fig 6A) and kidney weight (Fig 6B) in RC/RC mice, without significantly affecting overall body weight compared to vehicle-treated controls (Fig 6C). Pirfenidone did not affect cyst number (Fig 6D) or cystic index (cyst area/ total area of the kidney) (Fig 6E) in RC/RC kidneys. However, total cyst area and total kidney area were significantly reduced (Fig 6F,G). Pirfenidone treated RC/RC mice showed significantly lower blood urea nitrogen (BUN) levels (Fig 6H) and more intact renal parenchyma with less interstitial expansion compared to vehicle treated RC/RC mice (Fig 6I,J). Pirfenidone did not significantly alter mRNA expression of injury markers such as Kim-1 and Ngal in either genotype (Supplemental Fig 2A, B). In wildtype mice (WT) mice, pirfenidone treatment had no effect on renal morphology (Fig 6I,J), kidney-to-body weight ratio (Fig 6A), kidney weight (Fig 6B), or BUN (Fig 6H) compared to vehicle treatment.

## DISCUSSION

This study shows that pirfenidone effectively attenuates pro-fibrotic processes in human ADPKD myofibroblasts and renal fibrosis in the RC/RC mouse model of ADPKD. We found that myofibroblasts are a major source of ECM in human ADPKD kidneys, and that pirfenidone treatment inhibits ECM gene expression, cell proliferation, migration and contractility of primary human ADPKD renal myofibroblasts *in vitro*. In RC/RC mouse kidneys, pirfenidone treatment reduced myofibroblast abundance, ECM, pro-fibrotic cell signaling pathways and cyst growth compared to vehicle-treated controls. Together, these results highlight the central role of myofibroblasts in ADPKD-associated fibrosis and support the therapeutic potential of antifibrotic drugs like pirfenidone as complementary treatments alongside cyst-directed therapies.

Myofibroblasts are αSMA expressing, migratory cells capable of continuously producing copious amounts of ECM, and pro-fibrotic and pro-inflammatory factors that modify the interstitium in CKD kidneys and chronic diseases of most solid organs ^9, 31^. While myofibroblasts are scarce in the normal renal tubular microenvironment, they are abundant in ADPKD kidneys and often seen surrounding cysts. Our previous studies in ADPKD showed that renal cyst- lining epithelial cells stimulate myofibroblast differentiation, migration and proliferation *via* paracrine mechanisms, leading to their accumulation in the pericystic microenvironment ^10^. Remarkably, myofibroblast depletion in a mouse model of ADPKD not only reduced fibrosis, but also inhibited renal cyst growth, highlighting a unique pathogenic role for these cells in ADPKD ^11^.

In the current study, we evaluated the effect of pirfenidone on fibrosis in a mouse model of ADPKD, and on ECM production by human ADPKD renal myofibroblasts, to better reflect the human disease context. Given the limited characterization of ECM composition in ADPKD kidneys, we analyzed the publically available KIT snRNA-seq database, comparing normal and ADPKD human kidneys ^29^. We found that ADPKD fibroblasts are the major producers of multiple structural fibrous-ECM proteins, cell-adhesive ECM glycoproteins, ECM modulating enzymes and matricellular proteins when compared to other cell types. Moreover, compared to control normal fibroblasts, ADPKD fibroblasts exhibited elevated expression of numerous ECM and ECM-related genes, many of which have been implicated in the pathogenesis of kidney diseases. For instance, type I (COL1A1, COL1A2) and type III (COL3A1) collagens are known to promote interstitial fibrosis, ECM stiffening, myofibroblast activation and vascular remodeling in the kidneys ^32^. Moreover, Type IV collagens (COL4A1, COL4A2), which are normally integral to basement membranes can become pathogenic when mutated or overexpressed ^33^. Type V (COL5A1, COL5A2, COL5A3) and type VI (COL6A2, COL6A3) collagens are also implicated in ECM remodeling and progression of fibrosis ^34^. Similarly, periostin, fibrillin-1, fibronectin, thrombospondin-1, biglycan and versican contribute to fibrosis by modulating TGFβ signaling and matrix organization ^35-38^.

The ECM expression profile from KIT snRNA-seq data was corroborated in primary cultures of human ADPKD renal myofibroblasts. Importantly, pirfenidone treatment significantly reduced the expression of a broad panel of ECM-related genes in these human ADPKD myofibroblasts. Notably, while the majority of ECM and profibrotic genes were downregulated, COL6A2 exhibited a paradoxical increase. This finding may reflect compensatory or cell-type specific responses to antifibrotic therapy, and warrants further investigation. Beyond its effects on ECM gene expression, pirfenidone also significantly suppressed fundamental cellular behaviors of activated myofibroblasts including cell proliferation, migration, and matrix contraction. Collectively, these findings highlight the potential of pirfenidone to blunt both molecular and cellular drivers of fibrosis in ADPKD.

Consistent with the *in vitro* findings, pirfenidone treatment significantly reduced the renal myofibroblast population and ECM in RC/RC mouse kidneys. Mechanistically, the antifibrotic effect in RC/RC mouse kidneys was associated with inhibition of TGFβ/SMAD3 signaling, a key driver of myofibroblast activation. Supporting this mechanism, pirfenidone treatment of rat renal fibroblasts inhibited their TGFβ-stimulated differentiation to myofibroblasts *in vitro*. These results align with previous reports identifying inhibition of TGF-β signaling as a primary mechanism underlying pirfenidone’s antifibrotic action^17^. Additionally, pirfenidone downregulated mTOR signaling, suggesting a dual role in modulating fibrogenesis and cell proliferation. By inhibiting ECM production, myofibroblast proliferation and migration, and key signaling pathways such as TGFβ and mTOR, pirfenidone holds potential as a therapeutic strategy to mitigate renal fibrosis in ADPKD.

Although pirfenidone treatment did not significantly reduce cyst index or cyst number in RC/RC mice, it significantly decreased total kidney weight and cyst area, indicating suppression of kidney enlargement. Notably, pirfenidone’s antifibrotic effects occurred without evidence of nephrotoxicity, as renal injury markers (KIM1 and NGAL) remained stable and BUN levels improved. Pirfenidone showed favorable pharmacokinetics in humans including rapid absorption (Tmax: 0.33–1h) with a terminal half-life of 2-2.5 h, low inter-individual variability, and minimal accumulation with multiple dosing ^39^. Phase III trials and post-marketing study data for IPF also confirm pirfenidone’s safety and tolerability ^40, 41^. The common side effects included nausea, fatigue, weight loss and rash, with dose-dependent toxicities (GI, liver, skin) manageable through dose adjustment ^42^. In a previous study, we showed that nintedanib, a triple tyrosine kinase inhibitor that is also an FDA approved anti-fibrotic drug for IPF, reduced renal fibrosis and cyst growth, and preserved renal function in ADPKD ^20^. Although nintedanib has a broader range of action because of its kinase inhibition, it has more gastrointestinal side effects compared to pirfenidone ^43^.

Given that current treatments for ADPKD, such as tolvaptan, primarily target cyst growth pathways, pirfenidone may provide a complementary approach by addressing fibrotic remodeling. Future studies could assess the long-term effects of combination therapy on renal function decline and evaluate whether earlier intervention could enhance efficacy in inhibiting cyst development and in preventing or delaying fibrosis.

## Supplementary Material

Supplement 1

## Data Availability

All *in vivo* studies using mouse models were performed and tissues analyzed at the University of Kansas Medical Center. All data that was generated are provided in the figures in the main text or in Supplementary Material. All requests for data will be processed based on institutional policies for noncommercial research purposes and shared. Data sharing could require a data transfer agreement as determined by the University of Kansas Medical Center’s legal department.
