# Supplementary material for: Pirfenidone treatment attenuates fibrosis in autosomal dominant polycystic kidney disease
Source: bioRxiv. 2025 Aug 29:2025.08.25.672225. Preprint. [Version 1] doi: 10.1101/2025.08.25.672225 (PMC12407920; doi:10.1101/2025.08.25.672225)
Supplement: Supplement 1 [file media-1.pdf]

Supplemental Figure 1

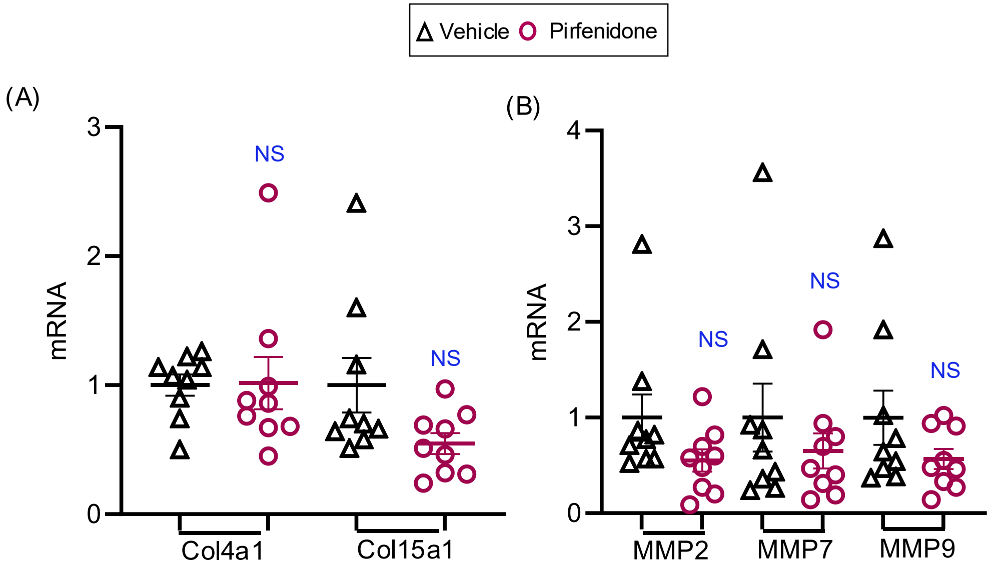

Supplemental Figure 2

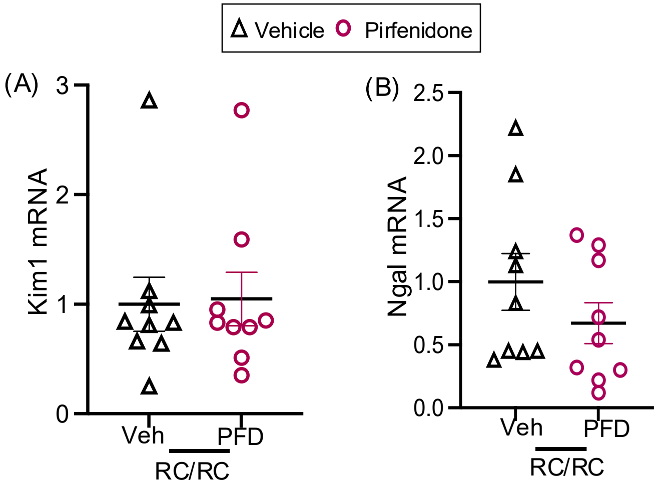

Suplemental Figure legends

Supplemental Figure 1: (A) Col4a1 and Col15a1 mRNA levels relative to 18S in RC/RC mouse renal tissues. (B) mRNA levels of MMPs in RC/RC mice renal tissues.

Supplemental Figure 2: (A) mRNA levels relative to 18S for Kim1 and (B) Ngal in mouse kidey tissue.

Supplemental Table-1

Human primer sequence used for QRT-PCR

	Gene Name	Primer Forward (F) and Reverse (R)
1.	MMP-2	F-AGCGAGTGGATGCCGCCTTTAA<br>R-CATTCCAGGCATCTGCGATGAG
2.	MMP-7	F-TCGGAGGAGATGCTCACTTCGA<br>R- GGATCAGAGGAATGTCCCATACC
3.	LAMA-4	F-GAGATGACTCTCTGCTGGACCT<br>R-AGTTCCAGGCAGCCAACAAAGC
4.	TIMP-1	F-GGAGAGTGTCTGCGGATACTTC<br>R-GCAGGTAGTGATGTGCAAGAGTC
5.	TIMP-2	F-ACCCTCTGTGACTTCATCGTGC<br>R-GGAGATGTAGCACGGGATCATG
6.	TIMP-3	F-TACCGAGGCTTCACCAAGATGC<br>R-CATCTTGCCATCATAGACGCGAC
8.	BGN	F-TTGAACCTGGAGCCTTCGATGG<br>R-TTGGAGTAGCGAAGCAGGTCCT
9.	LUM	F-AACATACCAACTGTCAATGAAAACC<br>R-TGCCATCCAAACGCAAATGCTTG
10.	FBN1	F-GGATACACAGGTGATGGCTTCAC<br>R-GTCGCATTCACAGCGGTATCCT
11.	VCAN	F-TTGGACCTCAGGCGCTTTCTAC<br>R-GGATGACCAATTACACTCAAATCAC
12.	NID1	F-ACATTGAGCCCTACACGGAGCT<br>R-GCCACTGGTAAGTGTAGATGCG
13.	ADAM12	F-ATGGCATCTGCCAGACTCACGA<br>R-GGAACTCTTCGAGACTTTGCCAC

14.	ADAM17	F-AACAGCGACTGCACGTTGAAGG<br>R-CTGTGCAGTAGGACACGCCTTT
15.	ADAM19	F-CGAGAAGGTGAATGTGGCAGGA<br>R-AGCTCTGACACTGGATCTTCCC
16.	THBS1	F-GCTGGAAATGTGGTGCTTGTCC<br>R-CTCCATTGTGGTTGAAGCAGGC
17.	THBS2	F-CAGTCTGAGCAAGTGTGACACC<br>R-TTGCAGAGACGGATGCGTGTGA
18.	THBS3	F-GGAAGGAGATGCCTGTGACAAC<br>R-GGTAGGATTGCTCATTTCAGGGC
19.	SPARC	F-TGCCTGATGAGACAGAGGTGGT<br>R-CTTCGGTTTCCTCTGCACCATC
20.	FBLN1	F-GTGGCTACCATCTCAACGAGGA<br>R-CTTGCATTCGCAGCGGAAACTG
21.	FBLN5	F-CTGCTGGATGACAACCGAAGCT<br>R-GATAAGGCTCCTCACAGCGGAT
22.	CCN1	F-GGAAAAGGCAGCTCACTGAAGC<br>R-GGAGATACCAGTTCCACAGGTC
23.	CCN2	F-CTTGCGAAGCTGACCTGGAAGA<br>R-CCGTCGGTACATACTCCACAGA
24.	Col1a1	F-TGACGTGATCTGTGACGAGAC<br>R-GGTTTCTTGGTCGGTGGGT
25.	Col1a2	F-CCTGGTGCTAAAGGAGAAAGAGG<br>R-ATCACCACGACTTCCAGCAGGA
26.	Col3a1	F- TGG TCT GCA AGG AAT GCC TGG A<br>R- TCT TTC CCT GGG ACA CCA TCA G
27.	Col4a1	F-TGTTGACGGCTTACCTGGAGAC<br>R-GGTAGACCAACTCCAGGCTCTC
28.	Col4a2	F-GGATAACAGGCGTGACTGGAGT<br>R-CTTTGCCACCAGGCAGTCCAAT
29.	Col5a1	F- GGAGATGATGGTCCCAAAGGCA<br>R- CCATCATCTCCTTTGTCACCAGG
30.	Col5a2	F- CAGGCTCCATAGGAATCAGAGG<br>R- CCAGCATTTCCTGCTTCTCCAG

31.	Col5a3	F-GAGAGGAGAACTGGGCTTCCAA<br>R-TAGAGGTCCCACTTCTCCTGTC
32.	Col6a2	F-CGTGGAGACTCAGGACAGCCA<br>R-CCTTTCAAGCCAAAGTCGCCTC
33.	Col6a3	F-CCTGGTGTAACTGATGCTGCCA<br>R-AAGATGGCGTCCACCTTGGACT
34.	Col12a1	F-CAGTGCCTGTAGTCAGCCTGAA<br>R-GGTCTTGTTGGCTCTGTGTCCT
35.	Col15a1	F- GGTGACACTGGTTTACCTGGCT<br>R-GCCTTTCCAGAGGAATGTCCTC
36.	POSTN	F- TGCCCAGCAGTTTTGCCCAT<br>R- CGTTGCTCTCCAAACCTCTA
37.	SERPINE1	F- CTCATCAGCCACTGGAAAGGCA<br>R- GACTCGTGAAGTCAGCCTGAAAC
38.	FBLN1	F- GTGGCTACCATCTCAACGAGGA<br>R- CTTGCATTCGCAGCGGAAACTG
39.	FBLN5	F- CTGCTGGATGACAACCGAAGCT<br>R- GATAAGGCTCCTCACAGCGGAT

Mouse primer sequence used for QRT-PCR

	Gene Name	Primer Forward (F) and Reverse (R)
1.	Adam12	F-TGCTACAACGGCATCTGCCAGA<br>R-GCTCTTGGAGTCTTTGCCACAG
2.	Adam17	F-TGTGAGCGGTGACCACGAGAAT<br>R-TTCATCCACCCTGGAGTTGCCA
3.	Adam19	F-GTGCCTCACTTACCAGGAACAG<br>R-GGACTGCACTTCCTGTATTGGC
4.	Thbs1	F-GGTAGCTGGAAATGTGGTGCGT<br>R-GCACCGATGTTCTCCGTTGTGA
5.	Thbs2	F-GTATGGAGGGAAGGACTGTGTC<br>R-ACTTGGCTCCAGGAAAACACGG
6.	SPARC	F-CACCTGGACTACATCGGACCAT<br>R-CTGCTTCTCAGTGAGGAGGTTG
7.	Lama4	F-CAGTTTGTCCTCTACCTCGGAAG<br>R-CTCACAGGCTTGGAATCCAGGA

8.	Bgn	F-TGAACCAGGAGCCTTTGATGGC<br>R-GTCCTCCAACTCAATAGCCTGG
9.	Vcan	F-GGACCAAGTTCCACCCTGACAT<br>R-CTTCACTGCAAGGTTCCTCTTCT
10.	Col1a1	F - AGA CAT GTT CAG CTT TGT GGAC<br>R - GCA GCT GAC TTC AGG GAT G
11.	Col1a2	F-TTCTGTGGGTCCTGCTGGGAAA<br>R-TTGTCACCTCGGATGCCTTGAG
12.	Col3a1	F - TCC CCT GGA ATC TGT GAA TC<br>R - TGA GTC GAA TTG GGG AGA AT
13.	Col4a1	F-CGGGTGTGAAAAGACCTATCGG<br>R-CTGGCATTCCTCTGACGCCTTT
14.	Col4a2	F-CGGGTGTGAAAAGACCTATCGG<br>R-CTGGCATTCCTCTGACGCCTTT
15.	Col5a1	F-AGATGGCATCCGAGGTCTGAAG<br>R-GACCTTCAGGACCATCTTCTCC
16.	Col5a3	F-GGCAAAGATGGTATTCCAGGACC<br>R-TGCTTCCTTTGTGACCAGGCATC
17.	Col5a2	F-GTGGCATAGGAGAGAAAGGTGC<br>R-GCCAACTAAGCCTCTAGGACCA
18.	Col6a1	F-GACACCTCTCAGTGTGCTCTGT<br>R-GCGATAAGCCTTGGCAGGAAATG
19.	Col6a3	F-CCTGGTGTAACTGATGCTGCCA<br>R-AAGATGGCGTCCACCTTGGACT
20.	Col8a1	F-GGAAATCCCACCTGTGCCAAGA<br>R-TCCTCTTGGTCCAGGTTCTCCA
21.	Col12a1	F-CAGCACCATGAATGTCGTCTGG<br>R-GGTCTTTGAGGATAGTGCTGGC
22.	Col15a1	F-ACACCCACAGTGACTCCCAAGA<br>R-TCCTCATTGCCCACGATGTCTC
23.	Col18a1	F-GTGACACTGGACCTCAAGGCTT<br>R-TTGTCTGAAGGAGGGTCCTGGT
24.	CCN1	F-GTGAAGTGCGTCCTTGTGGACA<br>R-CTTGACACTGGAGCATCCTGCA
25.	CCN2	F - GTGCCAGAACGCACACTG<br>R - CCCCGGTTACACTCCAAA

26.	aSMA	F - TCAGGGAGTAATGGTTGGAATG<br>R - GGTGATGATGCCGTGTTCTA
27.	18s	F - GTAACCCGTTGAACCCGA<br>R - CCATCCAATCGGTAGTAGCG
28.	TIMP1	F - CAAGGATGGACTCCTGGCACAT<br>R - TACTCGCCATCAGCGTTCCCAT
29.	TIMP2	F - AGCCAAAGCAGTGAGCGAGAAG<br>R - GCCGTGTAGATAAACTCGATGTC
30.	TIMP3	F- AGGATGCCTTCTGCAACTCCGA<br>R- GTGTAGACCAGAGTGCCAAAGG
31.	PAI-1	F - GAGGTGGAAAGAGCCAGATTTA<br>R- CCACTGAAGTAGAGGGCATTC
32.	MMP2	F - CAAGGATGGACTCCTGGCACAT<br>R - TACTCGCCATCAColGCGTTCCCAT
33.	MMP7	F - AGGTGTGGAGTGCCAGATGTTG<br>R - CCACTACGATCCGAGGTAAGTC
34.	MMP9	F - GCTGACTACGATAAGGACGGCA<br>R - TAGTGGTGCAGGCAGAGTAGGA
35.	HAI1	F- CTTCGTGAGGAAGAGTGCATGC<br><br>R-TCACACTCCAGGAAGCCATCGA
36.	AREG	F CAGAAGAATGGAAGAGTCAG<br>R CAGATATGCAGGGAGTCACC
37.	OPN	F TGAGAGCAATGAGCATTCCGATG<br>R CAGGGAGTTTCCATGAAGCCAC
38.	HE4	F - AGGTCAAGTCTCCACGAAGCCA<br><br>R - AGAACACTGGCTGTCCACCTGA

39.	NGAL	F - ATGTCACCTCCATCCTGGTCAG<br><br>R - GCCACTTGCACATTGTAGCTCTG
40.	KIM1	F-AAACCAGAGATTCCCACACG<br><br>R-GTCGTGGGTCTTCCTGTAGC
41.	FN1	F -ATGTGGACCCCTCCTGATAGT<br>R -GCCCAGTGATTTCAGCAAAGG
42.	ICAM1	F -CTTCCAGCTACCATCCCAAA<br>R -CTTCAGAGGCAGGAAACAGG
43.	IL-6	F- CTTCCATCCAGTTGCCTTCT<br>R- CTCCGACTTGTGAAGTGGTATAG
44.	IL-10	F-TTTGAATTCCCTGGGTGAGAA<br>R-ACAGGGGAGAAATCGATGACA
45.	TNF	F ACCCTCACACTCAGATCATCTTC R<br>TGGTGGTTTGCTACGACGT
46.	IFN	F -GGCCATCAGCAACAACATAAGCGT<br><br>R-TGGGTTGTTGACCTCAAACTTGGC
47.	CCL2	F -CTCGGACTGTGATGCCTTAAT<br>R- TGGATCCACACCTTGCATTTA
48.	CCL3	F-GAAGATTCCACGCCAATTCATC<br><br>R-GATCTGCCGGTTTCTCTTAGTC
49.	IL1b	F -TTGACGGACCCCAAAAGAT<br>R - GAAGCTGGATGCTCTCATCTG

50.	POSTN	F-CAAAGCACACAGTTACCTTTCCAGGG<br><br>R-GCAGGAAACCCACATTGCATGAGA
